# Category learning in a dynamic world

**DOI:** 10.3389/fpsyg.2015.00046

**Published:** 2015-01-30

**Authors:** Jessica S. Horst, Vanessa R. Simmering

**Affiliations:** ^1^Word Lab, School of Psychology, University of SussexBrighton, UK; ^2^Space Lab, Department of Psychology, University of Wisconsin—MadisonMadison, WI, USA

**Keywords:** dynamic systems, categorization, context, learning, cognitive development

Children develop in a real, messy world in which learning unfolds through time and space shared with others. Understanding how children develop in this complex environment will require a solid, theoretically-grounded understanding of how the child and environment interact—both within and beyond the laboratory. We as researchers understand the scientific value in testing children in carefully-controlled environments, but for our findings to have any impact on children's lives we must strive to understand how the processes we study in the lab operate in the real, busy environments in which children interact with peers and adults.

Categories, like children, do not exist in isolation. Consequently, category learning cannot be easily separated from the learning context—nor should it be. According to a systems perspective of cognition and development, categorization emerges as the product of multiple factors combining in time (Thelen and Smith, 1994). Here we illustrate this multicausality by considering the who, what, how, where and when of categorization. In this paper, we include many different types of behaviors under the umbrella term “categorization.” To be as inclusive as possible, we consider any case in which a participant responds to how stimuli may be grouped as evidence of category learning. This includes studies of word learning (generalizing a label from one instance to another), looking preferences (to stimuli from different familiar groups), and play (exclusively touching instances from the same category). The variety of tasks that relate to category learning exemplifies the importance of this fundamental process to a broad range of behaviors outside the lab.

You may notice in these examples that we have not included children's ages because, according to a systems view, research should not be about age *per se*. Our goal is not to create a catalog of milestones; our goal is to understand the cognitive mechanisms driving change. Therefore, we focus on the *developmental level* of the child. Obviously, age must be taken into account in experimental design because age is generally (but not perfectly) correlated with developmental level (e.g., appropriate motor responses differ for a 2-year-old vs. 2-month-old). Our point, however, is that we will learn more about category learning if we stop asking questions such as “how do prototype representations compare between 6 and 8 months of age?” and focus instead on the underlying learning mechanisms, e.g., “what causes prototype representations to change?”.

## Who is involved in learning

In the real world children learn through play and independent exploration (Hirsh-Pasek et al., 2009). However, in the lab children are seldom alone. This is important because children adjust their learning depending on who is providing information (e.g., the same or different experimenter, Goldenberg and Sandhofer, 2013; human or robot, O'Connell et al., 2009; mom or dad, Pancsofar and Vernon-Feagans, 2006). Children are also opportunistic and will look for any signal of what the right answer is. For example, children will track who is present when they hear a new word (e.g., Akhtar et al., 1996), whether the speaker has provided reliable information before (e.g., Jaswal and Neely, 2006) and whether a question is repeated (e.g., Samuel and Bryant, 1984). Thus, who is involved in learning matters both for learning in general and for category learning specifically. Moreover, who the *child* is also matters. For example, children with larger vocabularies more flexibly categorize the same stimuli on multiple dimensions (Ellis and Oakes, 2006; Horst et al., 2009); right-handed participants are more likely to associate “good” with right and “bad” with left (Casasanto and Henetz, 2012); and female participants learn phonologically-familiar novel words better than male participants do (Kaushanskaya et al., 2011, 2013).

## What is being categorized

All categories are not created equal: categories vary in complexity and within-category similarity (Sloutsky, 2010). Where children draw boundaries between categories is influenced by category (object) properties, including distinctive features (Hammer and Diesendruck, 2005), number of common features (Samuelson and Horst, 2007; Horst and Twomey, 2013), visual cues to animacy (Jones et al., 1991), the presence of category labels (Sloutsky and Fisher, 2004; Plunkett et al., 2008) and the presence of other objects (e.g., identical or non-identical exemplars Oakes and Ribar, 2005; Kovack-Lesh and Oakes, 2007).

In naturalistic environments, categories are often *ad hoc* and flexible (Barsalou, 1983). For example, the category “toys to pick up before bed” may be discussed every day, but each day it may include different items. Furthermore, the process of categorizing objects is not independent of the objects themselves: different objects may be more or less flexibly assigned to different categories depending on the context (Mareschal and Tan, 2007) and information available (Horst et al., 2009). Thus, in order to understand the *process* of categorization, researchers must ensure that the results they find in the lab are not too closely tied to the specific stimuli.

## Where categories are experienced and tested

We know that environment matters because there are significant effects of household chaos (Petrill et al., 2004), excessive classroom decorations (Fisher et al., 2014) and environmental noise (for a review see, Klatte et al., 2013) on children's cognition. Where a child lives impacts what social categories they learn and the category choices they make. For example, Black Xhosa children in South Africa prefer own-race faces if they live in a primarily Black township, but prefer higher-status race faces if they live in a racially diverse city (Shutts et al., 2011). Furthermore, where children live interacts with who they are: only infants from the statistically dominant race show “own-race” face preferences; infants from the minority race show no race preference (Bar-Haim et al., 2006).

In the lab, location matters both in terms of where the child is and where the stimuli are. For example, children are more likely to learn names for non-solid substances if introduced to the gooey items in a familiar highchair context (Perry et al., 2014). Children also benefit when learning and testing contexts are the same (Vlach and Sandhofer, 2011) and when stimuli locations are stable across naming instances (Samuelson et al., 2011).

## How and when category learning is probed

Different tasks support different types of category learning. For example, yes/no questions lead to a stronger shape bias than forced-choice questions (Samuelson et al., 2009), various types of feedback differentially affect learning categories with highly salient features vs. less salient features (Hammer et al., 2012) and highly variable category members facilitate category name generalization (Perry et al., 2010) whereas less variable category members facilitate category name retention (Twomey et al., 2014).

Categorization does not reflect static knowledge; rather, category learning unfolds over time and is a product of nested timescales. Children (and adults) are constantly learning: experimenters' distinction between learning vs. test trials is arbitrary with respect to the processes that operate within the task (McMurray et al., 2012). That is, learning continues even on test trials—in fact, participants may not realize the shift from learning to test trials. Consequently, different behaviors are observed depending on when during the categorization process category learning is assessed (Horst et al., 2005).

Category learning is a product of nested timescales including (a) the current moment (e.g., how similar the stimuli are on the current trial, Horst and Twomey, 2013), (b) the “just previous” past (e.g., what happens during the intertrial interval, Kovack-Lesh and Oakes, 2007; whether stimuli on the first test trial are novel or familiar, Schöner and Thelen, 2006; and trial order effects Wilkinson et al., 2003; Vlach et al., 2008) and (c) developmental history (e.g., vocabulary level, Ellis and Oakes, 2006; Horst et al., 2009; Perry and Samuelson, 2011). Because children's behavior is never solely the product of a single timescale it is impossible to create an experiment that taps only into category learning in the moment or only knowledge children brought to the lab. Each timescale is part of the time-behavior interaction. For example, Kovack-Lesh et al. (2008) demonstrated that 4-month-old children's ability to form a category of cats that excludes dogs is not due only to comparison (i.e., looking back-and-forth, in the moment) or having a pet at home (developmental history), but is due to the *interaction* of both factors.

## Unexpected influences

If researchers view categorization as static knowledge, then neither the when or how should matter. Many researchers hold this view, which purports experiments are designed to test what a child knows upon arrival at the lab: trial order and trial types are largely trivial. However, as the examples we provided collectively illustrate, the impact of seemingly “nuisance factors” are not just noise in the data; they are “unexpected influences” that change behavior in predictable ways and can provide insights into the underlying processes of learning and generalization. Small variations in what children experience during category learning can have dramatic impact on how they form categories (e.g., sequential vs. simultaneous presentation, Oakes and Ribar, 2005; Lawson, 2014) and differences in testing contexts can lead to indications of what has been learned (Cohen and Marks, 2002). Thus, it is vital to acknowledge the impact of such unexpected influences if we want to understand how categorization unfolds over time.

Subtle experimental design decisions, such as the number of test trials to include, may not seem theoretically significant, but they can have profound effects on children's behavior. As dozens of studies illustrate, “boring” factors like counterbalancing and stimuli choice during both learning and testing can have a profound effect on findings, including trial order (Wilkinson et al., 2003), how many targets (Axelsson and Horst, 2013) or competitors (Horst et al., 2010) are presented, or the color of the stimuli (Samuelson and Horst, 2007; Samuelson et al., 2007). For example, how broadly participants generalize a category label depends on where the exemplars are presented and if the exemplars are visible simultaneously (Spencer et al., 2011). In particular whether more or less diverse examples occur in the first block of trials influences later generalization (see Spencer et al., 2011, Supplementary Materials). A result like this sheds light onto the generalization process: deciding how broadly a category should be applied depends on the timing of experience with exemplars (see Oakes and Spalding, 1997; Samuelson and Horst, 2007, for similar findings).

Unexpected influences may not be of immediate theoretical interest to a given experimenter, but they are still often informative—even at times vital—to the underlying processes at work (e.g., the influence of novelty on children's selection is informative for understanding how prior memory influences current learning). Experimental designs should manipulate these types of factors as independent variables whenever possible. We recognize this can be impractical with populations that are costly to recruit, in which case such factors may be controlled for statistically, for example with item-level analyses.

## Outlook

Category learning unfolds across both space and time, and small differences at one moment (e.g., shared features among the stimuli; whether exemplars are identical) can create a ripple of effects on real behavior. Behavior emerges from the combination of many factors, including those not explicitly manipulated or controlled by the experimenter. To understand what causes developmental changes in behavior, we must also acknowledge and understand the processes through which these factors (sometimes unexpectedly) influence behavior in our tasks, including at short timescales. However, just as it is important to acknowledge these unexpected influences, we must not fail to see the forest for the trees. If a behavior such as category learning can only be captured in an ideal environment under carefully-controlled conditions, how much can we generalize to the contexts in which learning typically occurs? Theoretical accounts that neglect the rich influence of context in real time are too narrow to be applied outside the lab (Simmering and Perone, 2013). What we as researchers are ultimately trying to understand is how learning occurs in a real, cluttered world across time and a variety of contexts. Consequently, a solid, theoretically-grounded understanding of cognitive development will require understanding how the child (or adult) and environment interact. Only then will our theories be both comprehensive enough and sufficiently specific to reliably predict behavior and potentially intervene to prevent adverse outcomes.

### Conflict of interest statement

The authors declare that the research was conducted in the absence of any commercial or financial relationships that could be construed as a potential conflict of interest.
